# The asymmetric effects of 20 years of tariff reforms on Egyptian workers

**DOI:** 10.1007/s40888-020-00199-0

**Published:** 2020-09-28

**Authors:** Giorgia Giovannetti, Enrico Marvasi, Arianna Vivoli

**Affiliations:** 1 Università di Firenze, Via delle Pandette 32, 50127 Florence, Italy; 2 European University Institute, Via dei Roccettini 9, 50014 San Domenico di Fiesole, Italy

**Keywords:** Trade policy, Labour market, Wage, Inequality, Egypt, F13, F14, F16

## Abstract

After more than two decades of trade liberalization, faced with deep structural problems which were exacerbated by the 2008 financial crisis and culminated in the 2011 Spring Revolution and government change, in 2016 Egypt started to protect some sectors from foreign competition. This paper assesses how tariff reforms during the 1998–2018 period affected the Egyptian labour market by focusing on real wages and job stability (i.e. having a permanent position). The empirical analysis is carried out on worker-level data from the available four waves of Egyptian Labour Market Panel Survey (ELMPS), including the recently released 2018 wave. We find that higher tariff protection tends to worsen labour market conditions, both lowering real wages and decreasing the probability of finding a stable job. Furthermore, tariff changes show remarkable asymmetries. There is a negative and significant correlation between tariffs increases and real wages, while the positive impact of tariff reductions turns out to be negligible and insignificant. Our findings support the view that in Egypt protectionism hampered working conditions, contributing to inequality, while liberalizations did not improve nor deteriorate them.

## Introduction

The process of trade liberalization and market-oriented reforms that started in the early 1980s and intensified in the 1990s triggered developing countries’ integration in the global market. Their exports reached almost half of total world exports (44% of merchandise trade in 2019 and 34% of commercial service trade, WTO 2019) and their rapid economic growth, the so-called Great Convergence (Baldwin 2016), has often been attributed to the increase in trade openness.

The existing literature has inquired about the links between trade liberalization, growth and inequality. Earlier papers (see for all Dollar 1992; Edwards 1998) supported the idea that openness to trade was associated with better economic outcomes. Rodríguez and Rodrik (2000), on the other hand, claimed that “the relationship between trade policy and economic growth remains very much an open question” and “is far from having been settled on empirical grounds.” More recently the view that trade reforms generate substantial improvements in developing countries has again found empirical support (for a recent review, see Pavcnik 2017; Irwin 2019).

However, if globalization and international trade, accompanied by technological change, contributed to aggregate growth at the world level and reduced inequality between countries, their increased importance coincided with a raise in inequality within countries. These asymmetric effects of trade, already present in classical trade theory, have been emphasized in the “new new trade theory” models, where trade is associated with simultaneous destruction and creation of jobs, with changes in the wage distribution and in returns to skills (see for instance Helpman et al. 2010a). The mechanisms at work are different, for instance: changes in technology (such as the increasing use of computers), increase in the demand for skilled workers (and their wages) and trade-induced pro-competitive pressures in the market (that might help to avoid distortions). Due to increased inequality within-countries and other factors, including the economic and financial crises of 2008–2012, the liberalization process of the last decades seems now come to an end, with protectionist forces on the rise (WTO 2020).^1^

Egypt is no exception to this common trend: earlier liberalization was followed by a recent return to protectionism. In the last decades, structural reforms were implemented to modernize the country also in response to internal and external shocks. In the late 1970s, Egypt was the first among the Middle Eastern and North African (MENA) countries to adopt a trade liberalization policy.^2^ In the aftermath of the 1986 oil countershock, in the middle of a very severe economic crisis, the government implemented the economic reform and structural adjustment programs (SAP) and the structural adjustment loan (SAL) sponsored by the international financial institutions. These agreements recommended a strong orientation towards trade liberalization. For most of the 1980s and the 1990s, Egyptian tariffs, with few exceptions, were reduced. However, the dismantling of import-substitution policies in favour of the Structural Adjustment Program did not produce the expected economic results: while enhancing aggregate economic growth, these policies failed to include the different segments of the Egyptian labour market.^3^

In what follows, we focus on the labour market effects of trade policy reforms: the Egyptian trade liberalization provides an excellent setting in which to study these effects.

For several years, Egypt’s labour market was not able to absorb the abundant labour supply of educated (skilled) young adults. As a result, inequality increased (World Bank 2007).^4^ During the early 2000s, labour market indicators started to slightly improve, but the 2008 financial crisis and the 2011 Spring Revolutions took the country back to a situation of economic slowdown. In the attempt to stabilize the economy and reduce the political turmoil, starting from 2016, the Egyptian government implemented new structural reforms but this time opted for a return to protectionism. Overall, despite having been the first among the MENA countries to embrace trade liberalization policies, and member of the WTO since its start, Egypt remains one of the most protected countries in the region, with import tariffs well above the average. Moreover, Egyptian firms appear to be less integrated in the international market, with a lower percentage of exporting firms and more inefficiencies in clearing customs procedures. According to the Enabling Trade Index issued by the World Economic Forum, over time Egypt worsened from being the 87th (in 2008) to 116th (in 2016) amongst 136 countries for the ease of getting goods across the border, a serious deterioration.

Against this background, an evaluation of the success and the effects of first pro-market reforms and then protection is of primary interest.

We focus on tariff reforms during the last two decades and study their effects on the Egyptian labour market. Our main research questions are: how/to what extent did the tariff reforms affect wages and job stability of Egyptian workers? Did tariff increases have the same (opposite) impact as tariff liberalizations or is there an asymmetric effect? Did tariff reforms affect workers’ inequality by skill and gender?

Exploiting the available four waves of the Egypt Labour Market Panel Survey (ELMPS), we contribute to the debate with a 20-year view of Egyptian labour market dynamics. Our data include the early liberalization phases as well as the recent return to protectionist measures (in the recently released 2018 wave). The four waves of the ELMPS-1998, 2006, 2012 and 2018—offer a unique opportunity to investigate the long-term effects of the trade liberalization process on labour market outcomes as well as to explore the heterogeneity across different segments of the labour market. Moreover, thanks to the recent switch to protectionism, we are also able to test the potential existence of asymmetric effects of tariff increases versus tariff reductions.

Our results bring new evidence on the heterogeneous individual-level effects of two decades of tariff reforms in Egypt. We find that higher protection tends to worsen labour market conditions, both lowering real wages and decreasing the probability of finding a stable job (i.e. having a permanent formal contract). These results do not seem driven by aggregate trends and hold under several specifications, including panel and instrumental variables estimations. Furthermore, tariff changes show remarkable asymmetries. The negative and significant correlation between tariff increases and real wages does not hold for liberalizations: their impact turns out small or insignificant. A similar asymmetry applies to job stability: only tariff increases tend to have an effect, reducing job stability. Overall, our findings support the view that in Egypt protectionism hampered working conditions and inequality, while liberalizations did not improve nor deteriorate them.^5^

The paper is organized as follows. Section 2 reviews the existing literature. Section 3 describes Egypt’s tariff reforms and the Egyptian labour market for the period 2008–2018. Section 4 discusses data and methodology. Section 5 reports our empirical findings. Section 6 concludes.

## The effects of trade liberalizations on wages: a review of the literature

This paper connects to a broad literature studying the effect of trade liberalization on labour market outcomes.^6^ Until the 1990s, the model generally used to single out the possible link between trade and inequality was the Heckscher–Ohlin (H–O) model. Its most relevant prediction for the labour market comes from the Stolper–Samuelson theorem (S–S hereafter) stating that trade increases the real return to the factor that is relatively abundant and lowers the real return to the scarce factor. For a (low-skill) labour abundant developing country like Egypt, the S–S theorem predicts a negative correlation between tariffs and wages: a trade liberalization such as an import tariff reduction, by increasing specialization, will raise prices in the export sector and reduce them in the import sector. Consequently, workers (mostly unskilled) benefit from wage increases, while the remuneration of the factor used intensively in the import-sector declines (Baldwin 2008).

One problem with the H–O model is that it assumes perfect cross-sectoral factor mobility and therefore within-country factor price equalization. This is a limitation since it implies the existence of a single wage at the country level, which is very unlikely to hold when labour markets present rigidities. “The Heckscher–Ohlin model, the Ricardian model, and their cousins all assume that workers could switch industries without cost so that, if two industries are producing, they must offer the same wage to workers with the same ability. However, we know that this is not the way things work in the world. We have abundant evidence that there are frictions caused by switching industries, by moving across locations within a country, and by switching occupations. These frictions all imply that whether one gains or loses from a trade shock may be determined by one’s industry, location, occupation, or similar factors.” (McLaren 2017, p. 178).

In the case of Egypt, as we shall briefly see in the next section, the labour market is segmented and rigid. Hence, the standard H–O model is unlikely to provide an accurate description. Nonetheless, the mechanisms suggested by the S–S theorem remain appealing to explain the underlying long-run economic forces.

Within the standard trade models, the specific-factor model, which assumes imperfect cross-sector factor mobility or, in the basic version, that factors are fully sector-specific, may provide a more realistic description of the labour market. This model stresses that trade, beside affecting inequality through the skill premium (the S–S theorem), can also affect the industry wage premia intended as the “part of worker wages that cannot be explained by observable worker characteristics […], but can be attributed to workers’ industry affiliation” (p. 42, Goldberg and Pavcnik 2007).^7^ The main prediction of the model is that, if we assume labour as the immobile (and therefore specific) factor, it will lose from lower tariffs in the import competing firms but it will win in export-competing industries.

But, while the standard trade models provide useful insights, spurred by increased evidence and data availability, more recent models seem better suited to deal with the complexity of the real world and the faceted aspects of globalisation. Rooted on the Melitz’s model (2003) of the heterogenous firms and its developments, a strand of the theoretical literature provides new perspectives on the relation between trade and the labour market. In these models, trade liberalizations will push out of the market the least productive firms reallocating market shares towards the most efficient producers, thereby increasing average productivity.^8^ Provided that industry-level productivity enhancements are channelled to industry wages, these models also predict a negative correlation between tariffs and wages. The correlation is the same as in the H–O model, but the underlying mechanism is completely different.

Related to the “new new trade theory” approach, some papers highlight the effects of import competition and offshoring on wages and employment (e.g. Liu and Trefler 2008); some models analyse the effects of trade on inequality among workers doing the same job in the same industry, through heterogeneous-firms models or implicit-contracts models. Other authors assume a relationship between firm-specific wages and firm’s response to globalization; for instance, Helpman et al. (2010a) develop a new framework that incorporates firm and worker heterogeneity, search and matching frictions in the labour market, and screening of workers by firms. Their model finds that trade liberalization raises wage inequality.

In summary, the theoretical literature highlights different possible mechanisms to explain how changes in tariffs impact on wages and inequality. Which mechanism is likely to prevail is however inherently an empirical question, whose answer possibly depends on the economic context of the analysis. As a matter of fact, the available empirical evidence and, more closely related to this paper, the findings from studies that analyse individual and household data from developing countries are mixed. Some find no significant association between liberalization and industry wage premia (e.g. Feliciano 2001 for Mexico). Others find a positive relationship, primarily driven by technological change, through trade and imported inputs, favoring skilled workers (e.g. Attanasio et al. 2004, for Colombia) or driven by imperfect factor mobility (e.g. Kovak 2013, for Brazil). Lastly, in line with our results as well as with theoretical predictions from the above-mentioned models, some find that tariffs are negatively associated with industry wage premia (e.g. Kumar and Mishra 2008 for India; Murakami 2020, for Chile).^9^

Only a scant literature specifically focuses on Egypt. The relationship between tariff levels and wages is generally found to be negative, although differentiated by categories of workers. Said (2012) investigates the impacts of trade liberalization on job quality and wages in the manufacturing sector. Using both panel and quantile regressions for the 1998–2006 period, she finds a negative relationship between tariffs and hourly wages (measured in log), with a stronger impact on the working poor, the focus of the analysis. At the same time, further trade liberalization deteriorates the jobs quality and increases the precariousness of contracts. She also finds that gender and the private sector workers remain the most segregated categories in the labour market. Based on a cross-sectional analysis of the ELMPS 2006, Zaki (2014) finds that non-tariff measures and red tape costs have a strong impact on wage inequality. Workers employed in highly protected industries receive lower wages. Specifically, trade liberalization increases the regional, gender and skill wage gap. Aboushady et al. (2019), adopting the same methodology of Zaki (2014), analyse the effect of tariff, non-tariff measures and services restrictions on wage inequality on a cross-section of Egypt (2012), Jordan (2010) and Tunisia (2014). They find evidence that tariffs disproportionally and negatively affect blue-collar workers, whereas female workers seem to be more affected by non-tariff measures.

Overall, the wide literature on the relation between trade and the labour market highlights different possible mechanisms, some of them suggesting a negative correlation between tariffs and wages. While the empirical evidence reports mixed results, often country-specific, in the case of Egypt there seems to be a negative effect of trade liberalization on wages.

## Egypt’s tariff reforms and the labour market

Confronted by internal structural problems and challenged by the rapid evolution of the international context (with China that was emerging as the dominant world exporter), Egypt went through several policy changes regarding its tariff structure and the labour market, also following the ratification of the IMF structural programmes. We single out few stylized facts.

Despite a general trend of tariff reductions, mostly in line with other MENA countries, Egypt’s tariffs stood above the regional and world averages, shielding the already relatively inward-oriented domestic firms from import competition. Furthermore, the tariff liberalization trend was not smooth: it was characterized by ups and downs in tariff rates and by an increasing protection in the last years. Egyptian firms show a relatively low degree of internationalization, with only large firms exporting (see “Appendix” for more detailed information).

The global trend towards liberalization is likely to have been detrimental to Egypt, adding up to its structural problems: wage disparity between the public and private sector, existence of a large informal sector, and the inability of the labour demand to absorb educated young workers, since the productive structure is largely concentrated in agricultural and traditional sectors. Over time, Egypt implemented several labour market reforms, with mixed effects. For instance, the minimum wage was significantly increased. Yet, this was perceived as a temporary fix for deeper structural problems since the inherent complexity and low transparency of the wage system was not addressed (Amer 2015). To date, despite liberalizations and reforms, Egypt’s remains relatively protected with a complex and segmented labour market.^10^

### Tariff reforms

As several other low- and middle-income countries inside and outside the MENA region, in the 1960s and 1970s Egypt followed an import-substitution industrialization strategy. By the beginning of the 1980s, a combination of internal and external factors induced a reshaping of policies. The excess supply of oil stopped the inflow of foreign revenues that, in turn, lowered the ability of MENA governments to pursue both import-substitution policies and interventionist-redistributive commitment. Meanwhile, the world economy was entering a new paradigm with the collapse of the Communist bloc and the poor performance of inward-oriented economies relative to the extraordinary performances of the outward-oriented economies, especially from East Asia. Eventually, also MENA countries turned to stabilization and adjustment programs and followed a more outward-oriented approach.

Egypt was the first Middle Eastern North African country to adopt trade liberalization policies, already in the late 1970s. During the 1980s, the process of industrialization of the country suffered a tragic backlash. In 1986, Egypt was hit by the oil countershock and, facing a trade and fiscal deficit together with external debt of more than 150% of GDP, the country decided to address some of its structural problems with the implementation of the Economic Reform and Structural Adjustment programs sponsored by the international financial institutions. Following the new pro-market approach, from the 1990s Egypt started to sign bilateral and multilateral trade agreements. Within the African continent, Egypt became part of Common Market for Eastern and Southern Africa (COMESA) in 1998. In the same year, it also joined the Greater Arab Free Trade Area (GAFTA), thanks to which customs duties on imports from the 17 Arab countries reached zero in 2005 (Elkhafif et al. 2012). A similar trend applies to trade policies outside the African continent. Egypt joined the World Trade Organization from its start (1995), after having been a member of GATT since 1970. In 1995, on November 28th, together with the then 15 EU Member States and 12 Mediterranean Partner Countries (MPCs), Egypt was one of the co-signatories of the Barcelona Declaration to launch the Euro-Mediterranean Partnership Process. The Barcelona Process included the implementation of bilateral trade agreements and “the creation of a deep Euro-Mediterranean Free Trade Area, which aims at removing barriers to trade and investment between both the EU and Southern Mediterranean countries and between the Southern Mediterranean countries themselves”.^11^ The association agreement with the EU was signed by Egypt in 2001. In 2004, with Jordan, Tunisia and Morocco, Egypt joined the Agadir Agreement, establishing a free trade area amongst the Arab Mediterranean countries. Meanwhile, in 1999 Egypt had signed the Trade and Investment Framework Agreement with the US and the Qualified Industrial Zones Agreement with US and Israel. In 2005, Egypt signed a free trade agreement with Turkey. In August 2010, Egypt signed a preferential free trade agreement with MERCOSUR. The agreement entered into force after Argentina finalized its ratification process in July 2017. Lastly, in March 2018 Egypt signed the African Continental Free Trade Agreement (AfCFTA), with 44 signatories at that moment, became the largest free trade area in terms of ratifying countries since the formation of the WTO.^12^ This active involvement shows Egypt’s commitment towards trade liberalization.

**Fig. 1:**
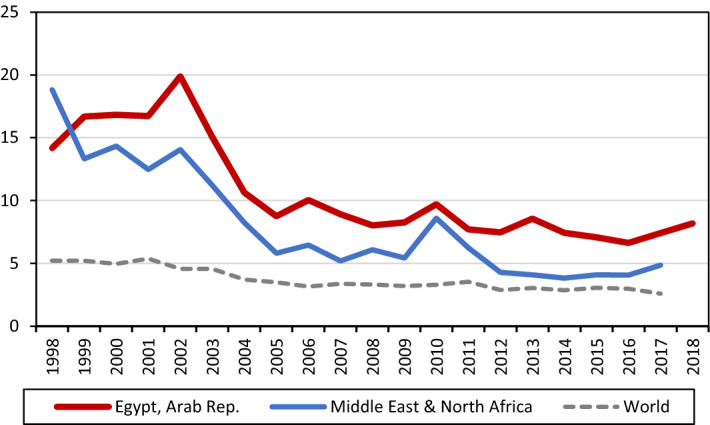
Trend in effectively applied tariff (AHS) for Egypt, MENA region and the world (weighted average, %) (Source: WITS database)

Two different phases describe these three decades of reforms: (i) a first wave, which started after the adoption of Structural Adjustment Programs in the mid-80s and lasted until the early 90s, and (ii) a second wave—analysed in this paper and depicted in Fig. 1—starting at the beginning of the new century, with the establishment of a new, more free-market oriented, approach (AlAzzawi 2016). Figure 1 shows a comparison of Egypt’s tariffs against the MENA countries and the World average. As mentioned above, despite liberalization, tariffs in Egypt have remained higher than in other MENA countries. During the period we study, two were the main objectives of tariff reductions. First, to rationalize the tariff structure and cut the tariff rates. Second, to simplify and reduce non-tariff barriers to trade (NTB). The number of tariff bands decreased from 27 to 6, and tariff lines went from 8000 to 6000. After the tariff reforms, in the manufacturing sector, nominal and effective tariff protection decreased from 21.3 to 12.1% and from 23.3 to 14% respectively after the reform in 2004 (Selwaness and Zaki 2015). Moreover, Egypt also lowered the number of products subject to NTBs. Some key industries, however, such as the food sector, had a high degree of non-tariff protection, while energy subsidies continued to be granted by the state.

If before 2011 the Egyptian government was committed towards a reduction in the number of tariff bands and to tariff cuts, in the aftermath of the 2011 Revolution, it changed direction. To face the new economic challenges, among which also a widening of the trade deficit, the Egyptian government raised import tariffs on a wide range of products, mainly non-agricultural goods (WTO 2018), including electronic devices, clothing, shoes, household appliances and plastics. Figure 1 shows a different reaction between Egypt and other MENA countries to the spring revolution: while Egypt increased tariffs, other countries did not. After a renewed commitment to liberalization (2013–16), in December 2016, the Egyptian government raised import tariffs for 364 tariff lines. In the Presidential Decree, the government described these goods as “provocative” or “unnecessary”. For most products, the increase was between 100 and 200%, while for the others it varied between 50, 125, 300, 500 and 700%.^13^ The lift on import tariffs aimed at cutting $49 bn trade deficit.^14^

In Table 1, we report the trends in sectoral tariffs for the period covered by our empirical analysis. Tariffs of almost all sectors have high correlation coefficients with the other sectors as well as with the aggregate trend (see the “Correlation” column of Table 1). Thus, despite some sectoral specificities especially in the level of protection, most sectors followed similar trends. Few sectors, nonetheless, display some peculiarities worth noting. The Hides and Skins sector shows a stable level in import tariffs (around 7%) with an extremely low but positive correlation coefficient with the general trend. Food Products, whose tariff dynamics move in the opposite direction relative to the general trend—is negatively correlated with all the other sectors—displays tariff rates growing from 20.9 to 100%. The textile-apparel sector shows a high correlation with the others over the entire period (see Table 1) and its dynamics are consistent with the country-wide figures; but it also displays important specificities due to sector-specific shocks that are worth mentioning. In 1998, the textile industry absorbed nearly 30% of total manufacturing and was one of the key sectors for the Egyptian economy (Magder 2005). But by the mid-2000s, when the Multi-Fibre Agreement ended and all quotas were removed, the sector, previously sheltered from international competition, faced severe challenges, as well as higher competition from East Asian economies. In the subsequent years, the share of textiles in Egypt's merchandise exports declined from 16.6% in 1995 to 4.5% in 2003 (WTO 2005) and employment almost halved from 22% in 2002 to 12% in 2010. Egypt’s trade policy for its apparel sector brought to a dispute in the international arena. Although Egypt agreed on removing its import ban on textile in 2002, as asked to do when it joined the WTO in 1995, at the same time it imposed dramatically high tariff rates. It has been estimated that Egypt increased its per item tariffs to 100–150%, a fact that led the US Trade Representative to take Egypt to task for breaking its commitment. Because of this, Egypt then agreed to fulfil its commitment to the WTO agreement and lowered its tariffs at the required level (Magder 2005).

**Table 1:** Egyptian tariffs, by sector, selected years and correlations with aggregate trend (Source: authors’ elaborations on WITS)

	Average 98–18	Min	Max	1998	2018	Correlation*
Animal	5.35	1.59	10.80	7.95	1.59	0.95
Chemicals	12.01	6.01	19.75	12.32	6.01	0.65
Food products	65.54	17.69	120.00	20.19	120.00	− 0.16
Footwear	32.80	24.21	46.09	38.58	45.95	0.57
Fuels	4.33	0.37	11.23	7.87	0.76	0.95
Hides and skins	24.15	13.09	43.71	29.69	42.51	0.07
Mach and elec	7.24	3.08	13.37	12.92	3.76	0.96
Metals	7.78	3.09	16.01	16.01	3.09	0.93
Minerals	2.15	0.11	9.50	9.50	0.11	0.88
Miscellaneous	9.97	5.46	14.70	13.32	7.33	0.93
Plastic or rubber	6.52	2.50	13.70	12.94	2.56	0.96
Stone and glass	7.45	2.88	18.35	18.35	8.31	0.29
Textiles and clothing	22.62	7.87	151.25	26.15	7.89	0.75
Transportation	30.39	19.05	44.07	44.07	19.51	0.71
Vegetable	2.28	0.20	6.08	6.08	0.38	0.93
Wood	6.96	2.12	13.93	11.89	2.33	0.94

*Correlations between each sector and the overall trend using MFN effectively applied rate (AHS)

In summary, given that the first wave of liberalisation followed the indications of the World Bank and the International Monetary Fund and the second wave was part of a more general reform program that had the objective of improving the business and investment climate, tariff reforms in Egypt were mostly driven by external forces or, at least, not generally modulated on sector-specific demands (Salem and Zaki 2019). The combination of co-movements of sectoral tariffs with the role of the multilateral negotiation to which Egypt took part over the years and with the rules to be followed when you are part of structural reform programmes, reduces possible concerns on the presence of sector-specific lobbying pressures or distortions and supports the view that tariff reforms in Egypt were largely exogenous (not imposed to provide differential protection, see Erten et al. (2019) for a similar argument for South Africa).

### Wages and the labour market

Egypt is signatory of some of the most important treaties worldwide, as the International Declaration of Human Rights (1948), the International Convention for Economic, Social and Cultural Rights (1966), and the International Labour Convention (1970), under which minimum wages are to be established and regularly updated within the rule of law. Moreover, Islamic law dictates that an employee should get his payment upon rendering the services and/or goods contracted (Abdelhamid and Baradei 2010). Despite the apparent ethical commitment, the Egyptian wage system has been often regarded as inequitable, complex and weakly enforceable (Lohmann 2010). The striking wage difference between public and private sector, for instance, signals the inefficient segmentation of the labour market. Estimates from the CAPMAS for 2018 puts public-sector average weekly wage at LE 1278, compared to LE 877 for the private sector. Complexity adds to the inequity of the system, making it opaque and easy to manipulate. The base salary upon which additions and pensions are calculated represents only 20% of the total wage earned by workers; the remaining 80% comes from a complicate system of special bonuses and incentives (Biltagy 2014).^15^

The national monthly minimum wage was set at LE 35 in 1984^16^ and remained at the same level until January 25th, 2011, the day the “Egyptian revolution” started. More than 25 years without any modification not surprisingly led to protests, with many employees fighting for a minimum wage reform. As a response to mass manifestations, in July 2012 the minimum monthly wage for all employees was raised to LE 700 and, 2 years later, in 2014, to LE 1200. Moreover, in March 2019, the President Al-Sisi raised it again, up to LE 2000.

These minimum wage increases, far from addressing the structural problems of Egypt’s labour market, resulted in a high wave of discontent throughout the nation. Through the minimum wage hike, the Al-Sisi administration was hoping to rapidly enhance the economic production and reduce social unrest. But Egypt’s structural issues had deep roots: heavy reliance on basic goods’ import, a stagnant private sector and a too large informal sector prevented the reform to have visible effects on production, causing instead an increase in wage gap between the public and private sectors. Indeed, wage legislation has been deemed as weak because it mainly affects the public sector and a small fraction of the private formal sector. To give an idea, according to El-Haddad (2019),^17^ the public sector shrank by 16% between 1998 and 2012, losing 40,000 jobs in the public administration between the 2006 and 2012 (World Bank 2014). However, the formal private sector was able to absorb only 4% of workers, implying that the majority of those losing a public sector job flew to the informal sector,^18^ which of course does not adhere to the minimum wage policy (in fact, 75% of the workers in the informal sector earn less than the minimum wage). Moreover, trade union membership is also weak and collective bargaining coverage in the private sector is limited, leaving Egyptian employees with a low bargaining power. Finally, the participation of women to the working force is particularly low. According to the ILO modelled estimates, female participation rates, at 25% in 2018 for the 15–64 age group, were the 11th lowest among 189 countries (Assaad et al. 2019).

## Data and methodology

### Worker-level data

We drew individual-level data from the Egypt Labour Market Panel Survey of 2018 (ELMPS 2018) (OAMDI 2019) carried out by the Economic Research Forum (ERF) with the Egypt’s Central Agency for Public Mobilization and Statistics (CAPMAS). The 2018 is the fourth round of a longitudinal survey, already administrated in 1998 and in 2006 and 2012. Covering exactly 20 years, the survey offers a unique opportunity to understand the long-term dynamics of the Egyptian labour market and its reactions to policy changes (in our case trade policy). The survey is composed of three sections: (i) households; (ii) individuals; (iii) income. The first section, the household questionnaire, is administrated only by the household’s head or by the head’s spouse. It contains questions on basic demographic characteristics of the members of the household, movements of the household’s members as well as questions regarding the ownership of assets and durable goods. The second section, the individual questionnaire, includes questions to which each person answers individually, concerning the educational background, employment and unemployment conditions and its reasons, average wage, job characteristics, mobility, job search activities, migration stories and a complete section on women’s work, their condition in the households and fertility. The 2018 wave dedicates more attention to measures of job stability, given the recent trends of the country towards higher precariousness. The third section encompasses all possible sort of income sources, from family-run agricultural and non-agricultural enterprises to transfers and remittances. The survey is representative at the national level. The ELMPS covers of the whole country, dividing it into six different macro- regions: Greater Cairo, Alexandria, Urban Lower Egypt, Urban Upper Egypt, Rural Lower Egypt and Rural Upper Egypt, with the only exception of the Frontier governorates. The final sample included 15,746 households and 61,231 individuals.

In order to measure the impact of trade liberalization reforms, we focused on workers in the traded sector. Therefore, our relevant sample includes 9704 households and 18,837 individuals. A large share of workers belongs to the agricultural sector, followed by manufacturing (see Table 11 in “Appendix”). Note that, by confining our attention to the traded sector, we may reduce the representativeness guaranteed by the whole ELMPS 18; but we are able to better investigate the impact of changes in tariffs on the workers that are directly affected by these changes.

Table 2 displays the descriptive socio-demographic statistics for our sample for each of the four waves. Real wage increased from 1998 until 2012, then decreased in 2018. Most of the workforce has primary or secondary degree (the average number of years of schooling being around six). Only about 6% (8% in 2012) of individuals have a university degree or above. Around the 90% of our sample is employed in unskilled (blue-collar) jobs and between 85 and 90% of the sample work in small or medium size firms. In 1998, 10% of the workforce was employed in public firms, and the percentage decreased further in the following years, in line with the stylized facts mentioned above. Lastly, the data confirm that the unionization of workers declined over time, passing from the 13% to the 5% of workers.

**Table 2:** Descriptive statistics for our working sample

	1998	2006	2012	2018
Real wage (ln)^a^	2.105	2.212	2.340	2.182
Male	0.491	0.509	0.618	0.524
Age	35.050	34.910	35.410	36.090
Urban	0.429	0.288	0.248	0.182
Married	0.614	0.656	0.730	0.750
Years of schooling	5.329	5.537	6.683	6.479
Primary-intermediate ed	0.936	0.936	0.917	0.933
Tertiary ed	0.064	0.064	0.083	0.067
Blue-collar	0.893	0.919	0.914	0.930
SME	0.857	0.929	0.840	0.867
POE	0.100	0.059	0.060	0.037
Trade union	0.134	0.082	0.061	0.053
N	4034	6927	6862	9181

^a^Wages are expressed as the natural logarithm of real hourly wages, expressed in 2018 Egyptian pounds using the Consumer Price Index

The wage differential between white and blue collars, measuring the skilled/unskilled wage gap, first widened, then stabilized for two waves, and then contracted in 2018. The 2018 fall was the consequence of the different developments: a small contraction in blue-collar wages versus a more pronounced fall of white- collar wages (Table 3). Male workers earn more than females: the gender wage gap increased between 1998 and 2006 to decrease afterwards (Table 4).

**Table 3:** Wages differential between white and blue collars across years

	1998	2006	2012	2018
Real wage (ln)	2.105	2.212	2.340	2.182
Blue-collar wage	1.976	2.073	2.233	2.136
White-collar wage	2.462	2.640	2.805	2.438
Wage gap	0.486	0.576	0.572	0.302
N	1325	1891	2619	2974

^a^Following Zaki (2014), we distinguish blue-collar workers (agriculture and production workers), from white-collar workers (clerical; technical and scientific; managers; sales and services)

^b^The wage gap is calculated as the difference between the natural log of average wages of blue- and white-collar workers

**Table 4:** Wages across gender across years

	1998	2006	2012	2018
Real wage (ln)	2.105	2.212	2.340	2.182
Female	1.829	1.817	2.179	2.019
Male	2.134	2.261	2.351	2.195
Gender wage gap	0.305	0.444	0.172	0.176
N	1325	1891	2619	2996

Workers with different individual attributes are not uniformly distributed across firms. Firms of different types, e.g. small vs. large and private vs. public, are characterized by heterogeneous workforce composition. Table 5 details the differences in earnings and in years of schooling between large and small and medium enterprises, with large firms hiring workers with a much better education level (years of schooling are almost double). Table 6 shows that publicly owned enterprises (POE) pay higher wages and require higher levels of education than private firms. Moreover, not surprisingly in the MENA area, public enterprises guarantee higher percentages of stable jobs.

**Table 5:** Wages and years of schooling across SME and large firms

	1998	2006	2012	2018
Large firms (above 100 employees)				
Real wage (ln)	2.224	2.296	2.592	2.309
Years of schooling	10.410	11.060	11.210	10.440
Small and medium firms				
Real wage (ln)	1.950	2.080	2.235	2.145
Years of schooling	5.953	5.074	6.119	6.335
N	868	3463	4770	5338

**Table 6:** Wages and years of schooling across private and public firms

	1998	2006	2012	2018
Private firms				
Real wage (ln)	1.973	2.105	2.266	2.146
Job stability^a^	0.859	0.913	0.782	0.752
Years of schooling	4.783	5.200	6.377	6.275
Publicly owned firms*				
Real wage (ln)	2.398	2.607	2.739	2.524
Job stability	0.955	0.917	0.921	0.935
Years of schooling	10.16	10.91	11.52	11.67
N	4029	6926	6860	9162

*Publicly owned enterprises include both government and state-owned enterprises

^a^Job stability is a dummy that takes the value of 1 when a worker reports to have a ‘permanent’ contract, whereas it takes the value of 0 for seasonal, casual and temporary contracts

### Tariff data

To assess the effects of trade liberalization processes, we match and merge the ELMPS worker-level data with sectoral level data those from the UN World Integrated Trade Solution (WITS) database. We use the trade-weighted average of the Most Favoured Nation ad valorem tariff rate (or its equivalent) disaggregated at the 3-digit level of the ISIC rev. Three classification (i.e. the maximum level of detail available in the ELMPS).^19^ In the following empirical analysis, the use of disaggregated tariff data guarantees a more precise picture of the policy changes as well as more variation. The choice of MFN tariffs rather than tariffs stemming from Preferential Trade Agreements (PTA) guarantees a broader, more representative, picture of Egypt’s policy orientation.^20^ PTA tariffs, in fact, being partner specific, might not provide an adequate picture of a country’s general attitude towards trade policy. Moreover, they may not reflect the true tariff barriers to trade even towards partner countries due to the effective use of preferences by firms. For instance, as argued by Cerdeiro and Nam (2018), firms gain access to PTA tariffs only if they can meet certain rules of origin and other requirements; however, this is not always the case, and preferences are not always applied. Sometimes firms can explicitly decide to forgo access to PTA tariffs if they deem that paying the non-PTA tariffs is cheaper and easier than complying with the PTA requirements.

**Fig. 2:**
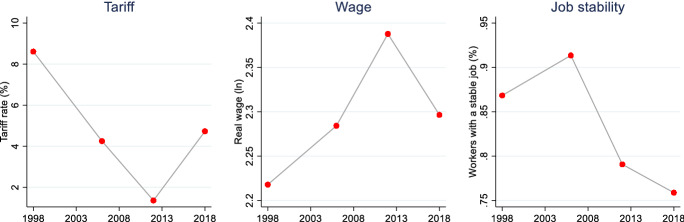
Trend in tariffs, wages, job stability and employment

Figure 2 summarizes the trends of tariffs, wages and share of workers with a permanent job position (job stability) for the workers included in our sample. Although, as discusses above, the sample includes traded sectors only, the overall trends are consistent with the general figures for Egypt showed in the first part of the paper.

### Methodology

We aim at testing the effect of tariff changes on job market outcomes. Our empirical specification follows Galiani and Porto (2010). Their approach consists of regressing individual wages for the entire time span on both individual-level characteristics and industry-level characteristics (in our case sectoral tariffs) directly in one stage.^21^ We employ the following baseline specifications:

$${y}_{i,t}=\alpha +\beta {\tau }_{t-1}+{{\varvec{x}}}_{i,t}^{\boldsymbol{^{\prime}}}{\varvec{\gamma}}+ {\varepsilon }_{i,t}$$

$$\Delta {y}_{i,t}=\alpha +\beta\Delta {\tau }_{t-1}+{{\varvec{x}}}_{i,t}^{\boldsymbol{^{\prime}}}{\varvec{\gamma}}+ {\varepsilon }_{i,t}$$

where ${y}_{i,t}$ is the job market outcome dependent variable for worker (or individual) *i* in year *t* = {1998, 2006, 2012, 2018}. We use two job market outcome variables: (i) the natural logarithm of the real wage; (ii) a dummy equal to 1 if the worker has a stable job. Our variable of interest, $\tau$, is the natural logarithm of the tariff rate^22^ defined at the 3-digit sector level in which the worker operates in the last 3 months before the interview. We consider tariffs in levels lagged by 1 year (i.e. the year before the survey) to reduce simultaneity concerns and to take into account the fact that tariff changes may display their effect after some time. Since the different surveys are not equally spaced over the observed time span, (wage and tariff) changes can only be defined over the observed period of *k* years from one survey wave to the other. To tackle this issue, we annualized the change by dividing it by *k*. The control variables vector, ${{\varvec{x}}}_{i,t}$, includes: (i) basic individual attributes such as age, gender, married status, whether they live in urban setting; (ii) worker’s characteristics as skill, proxied either by blue-collar status, education attainment, or years of schooling; participation to a trade union and the formal/informal status of the worker; (iii) firm’s characteristics such as size (small and medium = 1–100, large = above 100 employees), publicly owned enterprise (POE) and foreign-owned firms.^23^ Based on the above specifications, as explained more in detail in the next section, we estimate a pooled cross-section OLS, a fixed effect panel OLS and a first-difference OLS. Additionally, depending on the specification, we include: year fixed effects; sector fixed effects (at 2- or 3-digit level); Egypt’s governorate fixed effects; governorate-sector fixed effects; worker fixed effects (i.e. a panel regression).^24^ The term ε_*i*_, denotes the error.

Since the observation unit is the individual worker but tariffs are observed for the 3-digit sectors in which the workers operate, the source of variation in the worker-level tariff includes both within-sector changes due to trade policy decisions (i.e. tariff changes) as well as between-sector changes due to workers (or firms) moving across sectors, which of course is not directly due to the trade policy. In our data, this is an issue of little practical relevance as the share of workers who explicitly report to have changed sector is negligible (below 6%). Hence, we do not address the issue in this paper.

Building on the above specifications, we also investigate the possible asymmetric effects of tariff increases and decreases. To this aim, we decompose tariff changes and create a tariff increase dummy, ${I}_{t-1}$, defined over the *k*-periods between consecutive surveys and employ the following specification:

$$\Delta {y}_{i,t}=\alpha +{\beta }^{+}\left|\Delta {\tau }_{t-1}\right|{I}_{t-1}+{\beta }^{-}\left|\Delta {\tau }_{t-1}\right|\left(1-{I}_{t-1}\right)+{{\varvec{x}}}_{i,t}^{\boldsymbol{^{\prime}}}{\varvec{\gamma}}+ {\varepsilon }_{i,t}$$

Note that this corresponds to a model with a single interaction term:

$$\Delta {y}_{i,t}=\alpha +\beta \left|\Delta {\tau }_{t-1}\right|+\delta \left|\Delta {\tau }_{t-1}\right|{I}_{t-1}+{{\varvec{x}}}_{i,t}^{\prime}{\varvec{\gamma}}+{\varepsilon }_{i,t}$$

where $\beta ={\beta }^{-}$ and $\delta ={\beta }^{+}-{\beta }^{-}$. In this case, however, the first specification makes it easier to read the coefficients since ${\beta }^{+}$ is the effect of a 1% tariff increase and ${\beta }^{-}$ is the effect of a 1% tariff decrease.

Another way to capture this asymmetry is to allow for nonlinearities, for instance by including a quadratic term in the regression:

$$\Delta {y}_{i,t}=\alpha +\beta\Delta {\tau }_{t-1}+\delta {\left(\Delta {\tau }_{t-1}\right)}^{2}+{{\varvec{x}}}_{i,t}^{\boldsymbol{^{\prime}}}{\varvec{\gamma}}+ {\varepsilon }_{i,t}$$

where the $\delta$ coefficient governs the curvature of the equation. In this case, however, interpreting the asymmetry from the regression output is not immediate and calculating the margins or the marginal effects is preferable.

We employ a similar approach to investigate possible asymmetries due to skill and gender. In this case, however, since we are primarily interested in the existence of a gap, i.e. whether δ ≷ 0, the single-interaction specification is preferable:

$$\Delta {y}_{i,t}=\alpha +\beta\Delta {\tau }_{t-1}+\delta\Delta {\tau }_{t-1}{UnSkill}_{i}+{{\varvec{x}}}_{i,t}^{\prime}{\varvec{\gamma}}+{\varepsilon }_{i,t}$$

$$\Delta {y}_{i,t}=\alpha +\beta\Delta {\tau }_{t-1}+\delta\Delta {\tau }_{t-1}{Male}_{i}+{{\varvec{x}}}_{i,t}^{\prime}{\varvec{\gamma}}+{\varepsilon }_{i,t}$$

where ${UnSkill}_{i}$ is a dummy equal to one if the worker is unskilled, proxied by blue-collar status, and ${Male}_{i}$ is a male dummy.

### Endogeneity and identification strategy

As explained in the previous section, we investigate the relationship between tariffs and labour outcomes adopting several specifications, controlling for several observable characteristics of the workers as well as for different combinations of fixed effects capturing unobserved factors. As it will emerge clearly in the next section, our analysis aims at checking the existence of a negative correlation between tariffs and wages in Egypt for the 1998–2018 period. Caution is required in the interpretation of our results as causal effects. A major concern, widely discussed in the literature, regards the endogeneity of trade policy measures. Market labour conditions can be easily thought to influence the trade policy implemented by governments and trade barriers are often raised in the attempt to protect firms and workers from import competition. A vast theoretical and empirical literature investigated the political economy of trade policy and the possible role played by industry lobbies and interest groups in the determination of trade policy outcomes. Due to lobbying, governments may deviate from the socially optimal trade policy and partially accommodate demands from politically organized sectors. The equilibrium trade policy entails different levels of protection in the different sectors contingent on their political contributions.^25^

We fully recognize these concerns. Yet, in the case of Egypt there are several elements suggesting that trade policy reforms are likely to be driven by external factors. First, as mentioned in the first part of the paper, the liberalization waves essentially followed the indications of the international institutions as the World Bank and the International Monetary Fund (first) and the WTO (later), or a countrywide attempt to internationalize the economy, thus making tariffs in Egypt “less likely to be endogenous than those in other countries” (Salem and Zaki 2019). Second, and consistently with the previous point, tariff trends were very similar across industries as shown by the correlations in Table 1, and this co-movements of tariffs, in turn, suggests an absence of interference or lobbying from the private sector (see Erten et al. 2019).

However, to address the potential endogeneity problem linked with the reduction of tariffs, we instrument tariff changes with lagged tariff levels (i.e. previous survey year) and pre-liberalization tariffs (i.e. before the starting of the second wave of tariff reforms). This strategy is in line with some seminal papers in this field as Goldberg and Pavcnik (2005) and Bigsten et al. (2016); moreover, it seems appropriate given the history of tariff reforms in Egypt, as discussed above. The exclusion restriction requires that our instruments, past tariff levels, does not directly contribute to changes in workers’ wages (and probability of finding a stable job) several years after, as seems plausible, while they instead influence subsequent tariff changes. This is in fact what one would expect in a generalized tariff reform as the one that took place in Egypt. The negative correlation between tariffs and subsequent tariff changes (computed as − 0.52 meaning that tariff reductions were larger in more protected sectors) supports this exclusion restriction.

## Econometric analysis

### The effect of tariffs on wages and job stability

Let us start from a baseline specification in logarithms to which we add several relevant control variables as well as different combinations of fixed effects in order to absorb different possible sources of unobserved variability. We report the main results in Table 7.^26^ The first four specifications (columns 1–4) are cross-sectional pooled OLS estimations; below the coefficients, we report robust standard errors clustered at the sectoral level to consider possible correlations within industries. Model 1 includes our main explanatory variables and all the controls as well as fixed effects at the governorate level and at the 2-digit sector level, to capture industry-specific, time-invariant fixed characteristics. In Model 2 we add fixed effects at the governorate-sector level. The following two models mirror the previous ones, but at a more disaggregated level. In Model 3, we include 3-digit industry fixed effects and in Model 4 fixed effects at the governorate-3-digit sector level. Lastly, Model 5 reports the results of the panel estimation with worker-level fixed effects and robust standard errors, thus controlling for all the observable and unobservable time-invariant individual-specific characteristics as sex, individual talent etc.^27^

**Table 7:** Baseline regressions for real wage in levels

Variables	(1)	(2)	(3)	(4)	(5)
	Real wage (ln)	Real wage (ln)	Real wage (ln)	Real wage (ln)	Real wage (ln)
Tariff_t-1_ (ln)	− 0.064***	− 0.071***	− 0.104***	− 0.104***	− s0.053***
	(0.023)	(0.025)	(0.026)	(0.029)	(0.016)
Age	0.004**	0.004**	0.004***	0.004**	0.008***
	(0.001)	(0.001)	(0.001)	(0.002)	(0.002)
Male	0.223***	0.200***	0.221***	0.202***	
	(0.027)	(0.022)	(0.025)	(0.020)	
Urban	0.024	0.034	0.025	0.022	0.473**
	(0.026)	(0.027)	(0.024)	(0.025)	(0.201)
Married	0.151***	0.156***	0.150***	0.149***	0.174***
	(0.027)	(0.031)	(0.028)	(0.032)	(0.040)
Formal	0.117***	0.109***	0.114***	0.115***	0.053
	(0.020)	(0.022)	(0.020)	(0.024)	(0.050)
SME	− 0.003	0.023	− 0.015	0.009	0.019
	(0.030)	(0.030)	(0.033)	(0.032)	(0.046)
POE	0.024	0.037	0.030	0.017	− 0.018
	(0.032)	(0.038)	(0.030)	(0.041)	(0.081)
Union	0.292***	0.276***	0.289***	0.286***	0.131**
	(0.031)	(0.031)	(0.028)	(0.027)	(0.055)
Foreign ownership	0.667**	0.722**	0.694***	1.059***	0.911***
	(0.246)	(0.287)	(0.248)	(0.193)	(0.327)
Blue collar	− 0.143***	− 0.131***	− 0.144***	− 0.127***	− 0.043
	(0.018)	(0.021)	(0.021)	(0.022)	(0.060)
Constant	2.101***	2.251***	2.168***	2.300***	1.732***
	(0.111)	(0.124)	(0.112)	(0.122)	(0.121)
Observations	7111	7111	7111	7111	7111
R-squared	0.230	0.316	0.236	0.331	0.067
Governorate f.e. [22]	Yes	Yes	Yes	Yes	No
2-Digit sector f.e. [38]	Yes	Yes	No	No	No
3-Digit sector f.e. [64]	No	No	Yes	Yes	No
Gov-sector f.e. [576/724]	No	Yes	No	Yes	No
Worker f.e	No	No	No	No	Yes
Number of id					5667

Robust standard errors in parentheses. S.E. are clustered at the 3-digit sector level in models (1) to (4)

*p < 0.1; **p < 0.05; ***p < 0.01

Table 7 shows that the coefficient of tariff is negative and significant throughout all the specifications suggesting that tariffs are negatively correlated with real wages. The numerical size of the coefficient remains quite stable across the different specifications: a 1% tariff increase is associated with real wage decrease that span from 0.05 to 0.1%, other things equal.^28^ These numbers are consistent with similar studies on Egypt as Aboushady et al. (2019) and Zaki (2014). The negative correlation between tariffs and wages seems a specific characteristic of the Egyptian economy as results from other countries, such as for instance for Argentina in the study by Galiani and Porto (2010), reach opposite results, suggesting that the effect of tariffs is ultimately an empirical question. In our regressions, the coefficients of the control variables are in line with expectations. The effect of age and of being married is positive and significant. We detect the presence of both a skill and a gender wage gap: on average, a male worker earns around a 20% higher wage than a female worker and a blue-collar worker is penalized with a wage that is about 13% lower than a white-collar worker; furthermore, working in the formal sector assures almost a 11% higher wage. On the other hand, we find no clear evidence in favour of an urban bias (the urban dummy coefficient is positive but insignificant in all specifications but the panel). Members of trade unions display a wage premium, as well as foreign-owned enterprise workers, while workers in small and medium firms and in private firms do not seem penalized.

Results from Table 7 control for several time invariant factors. However, these results might be influenced by aggregate trends and labour market dynamics. To control for this possibility and exclude that our results are driven by macro-trends, we run our estimations controlling also for time effects. Fully controlling for both time and disaggregated sector fixed effects is, of course, the preferable empirical strategy, but it requires richer data with several survey waves and a more balanced panel (e.g. the study by Galiani and Porto (2010), uses almost 30 years of data coming from 40 surveys, with over 29,000 observations). Having just four survey waves and a highly unbalanced panel, also due the long time span between survey waves, we can investigate the time dimension only by reducing the sectoral detail. Specifically, we check our results including in the model year fixed effects, a linear trend, governorate-specific and sector-specific linear trends, together with sector dummies (in this case at 1-digit level). We report results in Table 16 in “Appendix”. Estimates are robust to the inclusion of time effects and the negative effect of tariffs remains significant in all specifications, thus confirming that tariffs acted as a specific channel other than the exchange rate and other macroeconomic changes. To further control for omitted time-invariant characteristics, we also investigate the effect of tariff increases on wage increases. The parameter of interest keeps its interpretation of elasticity; hence, its economic sense is the same as in the previous models. But now the addition of control variables captures the fact that the different categories of workers may experience different rates of growth of their wages rather than different wage levels. Unfortunately, running the models in differences implies a reduction in the number of observations due to the panel being unbalanced, and thus entails some loss of information. Despite the exclusion of workers observed only in 1 year (or not observed in consecutive years) may introduce a selection bias into the analysis, results hold. Model 1 and Model 2 of Table 8 include fixed effects at the governorate and at 3-digit sector level. In line with our previous findings, tariff changes are negatively correlated with real wage changes, other things equal. The numerical size of the coefficient (− 0.08–0.09) is also comparable with previous estimates. Due to the loss of observations with respect to the models in levels, Model 2, which replicates Model 1, excludes some controls. Again, results are robust. Lastly, note that the fact that most of the control variables are now statistically insignificant—with the exception of age, foreign ownership and unionization—is not in contrast with our previous estimates since the interpretation of the control variables is now different. For instance, foreign-owned firms’ workers are found to benefit from both higher wages (models in levels) and higher wage growth (models in differences); older workers, instead, display higher wages but the age-related gains slow down over time.

**Table 8:** First differences and Instrumental Variable regression for changes in wages

Variables	(1) OLS	(2) OLS	(3) IV	(4) IV
	Δ Wage	Δ Wage	Δ Wage	Δ Wage
Δ Tariff_t-1_ (ln)	− 0.091***	− 0.084***	− 0.112***	− 0.105***
	(0.024)	(0.024)	(0.021)	(0.020)
Age	− 0.001***	− 0.001***	− 0.001***	− 0.001***
	(0.000)	(0.000)	(0.001)	(0.001)
Sex	0.017	0.012	0.007	0.004
	(0.011)	(0.010)	(0.013)	(0.012)
Urban	0.009	0.009	0.013	0.012
	(0.008)	(0.008)	(0.009)	(0.009)
Married	0.002	0.005	0.006	0.008
	(0.008)	(0.009)	(0.009)	(0.010)
Formal	− 0.000	0.006	− 0.008	0.002
	(0.009)	(0.008)	(0.008)	(0.008)
SME	0.001		− 0.003	
	(0.008)		(0.009)	
POE	− 0.005	0.008	− 0.003	0.010
	(0.013)	(0.011)	(0.012)	(0.011)
Union	0.034***		0.037***	
	(0.011)		(0.011)	
Foreign ownership	0.152**	0.135**	0.077***	0.058***
	(0.062)	(0.065)	(0.015)	(0.017)
Blue collar	0.002	− 0.005	− 0.001	− 0.008
	(0.008)	(0.007)	(0.010)	(0.008)
Constant	0.048**	0.057***	0.074***	0.076***
	(0.023)	(0.018)	(0.022)	(0.020)
Observations	1525	1720	1334	1505
R-squared	0.097	0.085	0.104	0.089
Governorate f.e. [22]	Yes	Yes	Yes	Yes
3-Digit sector f.e. [64]	Yes	Yes	Yes	Yes

Robust standard errors in parentheses. S.E. are clustered at the 3-digit sector level. IV first stage: Under-identification test: Chi-squared p-value of 0.0158; weak instruments F-statistics 444.72

*p < 0.1; **p < 0.05; ***p < 0.01

Model 3 and 4 of Table 8 report results of the second stage of the IV regressions, where changes in tariffs have been instrumented with lagged levels of tariffs and pre-liberalization tariffs. Again, our results hold, with the coefficient of tariffs negative and significant. Under-identification and weak instruments tests do not reject our instruments choice. With a Chi-squared p-value of 0.0158, the under-identification test confirms our model; moreover, the high numerical value of the F-statistics of the first stage 10 (444.72) suggests that our analysis does not seem to suffer from weak instruments.

Lastly, we did some further checks on our results by investigating possible heterogeneity by sector and by subperiods. We report the full tables of results in “Appendix” for space reasons. For what concerns sectors, we report results for food and textile, chosen because of their peculiarities: the food sector was the only one following a different trend, while the textile sector was hit by a major external shock during the period of analysis: the end of the Multi-Fibre Agreement. Excluding the food and the textile sectors as well as adding tariff-sector interactions confirms our results, even though some degree of sectoral heterogeneity seems to exist, especially for the food sector. Finally, focusing on subperiods also produces coherent results.

Let us now investigate the effects of tariff reforms on job stability of workers, that is on the probability of having a permanent position. Reading together our main results on wages with those on job stability allows us to better understand the possible effects of the tariff reforms in Egypt. As our previous findings suggest, tariff increases are associated with wage reductions. If obtaining a permanent position becomes more likely, it may partly compensate the workers thus attenuating the negative effects due to higher tariffs. If, instead, wage reductions come together with increased job instability, then the position of workers worsens even more.

Our dependent variable is now a job stability dummy. The econometric specifications follow the previous analysis. Note that job stability is observed only for subjects who work, as in the wage regressions; however, the number of observations is larger, allowing us to better exploit the data. Since the dependent variable is binary, our results refer to the probability to have a permanent position. We present the results from linear probability models (LPM) estimated through OLS.^29^ We report all the main results in Table 9. Model 1 include tariffs in levels, while in Model 2 we use changes in tariffs. Both Models have governorate and 3-digit industry fixed effects as well as robust standard errors clustered for sectors. Model 3 is a worker-fixed effects panel regression with robust standard error and, Model 4, reports the IV estimation, with pre-liberalization and lagged tariff levels as instruments. In all specifications, tariffs are negatively correlated with job stability.

**Table 9:** Main results for job stability

Variables	(1) OLS	(2) OLS	(3) OLS	(4) IV
	Job stability	Job stability	Job stability	Job stability
Tariff_t-1_ (ln)	− 0.106		− 0.074***	
	(0.090)		(0.011)	
Δ Tariff_t-1_ (ln)		− 0.769**		− 0.961***
		(0.300)		(0.281)
Age	0.005***	0.006***	− 0.014***	0.006***
	(0.001)	(0.002)	(0.002)	(0.001)
Sex	− 0.101**	− 0.104		− 0.004
	(0.040)	(0.079)		(0.027)
Urban	− 0.003	0.020	− 0.050	0.031
	(0.022)	(0.033)	(0.097)	(0.035)
Married	− 0.009	0.034*	0.003	0.015
	(0.041)	(0.018)	(0.027)	(0.012)
Formal	0.065	0.013	− 0.003	− 0.020
	(0.039)	(0.030)	(0.034)	(0.030)
SME	0.048**	0.065*	0.114**	0.055
	(0.023)	(0.035)	(0.048)	(0.037)
POE	0.094***	0.095***	0.051	0.099***
	(0.018)	(0.026)	(0.035)	(0.022)
Union	0.182	0.486***	0.924***	0.494***
	(0.177)	(0.103)	(0.048)	(0.042)
Foreign ownership	0.005	− 0.041	− 0.079**	− 0.060
	(0.020)	(0.034)	(0.036)	(0.038)
Blue collar	0.200***	0.124***	0.129***	0.157***
	(0.023)	(0.036)	(0.036)	(0.025)
Constant	0.565***	0.431**	1.373***	0.394**
	(0.071)	(0.190)	(0.081)	(0.158)
Observations	11,713	3706	11,713	3176
R-squared	0.143	0.204	0.056	0.216
Governorate f.e. [22]	Yes	Yes	No	Yes
3-Digit sector f.e. [64]	Yes	Yes	No	Yes
Worker f.e	No	No	Yes	Yes
Number of id			8863	

Robust standard errors in parentheses. S.E. are clustered at the 3-digit sector level

*p < 0.1; **p < 0.05; ***p < 0.01

### Asymmetric effects and inequality

Our results indicate a negative effect of tariffs on wages of Egyptian workers. This effect, however, represents an average obtained by holding constant several observable and unobservable factors. In this section we disentangle the possible existence of some types of asymmetries and unequal effects by type of workers. Specifically, we focus on the potential asymmetry between tariff increases and decreases, and on unequal effects by workers’ skill and gender.

**Fig. 3:**
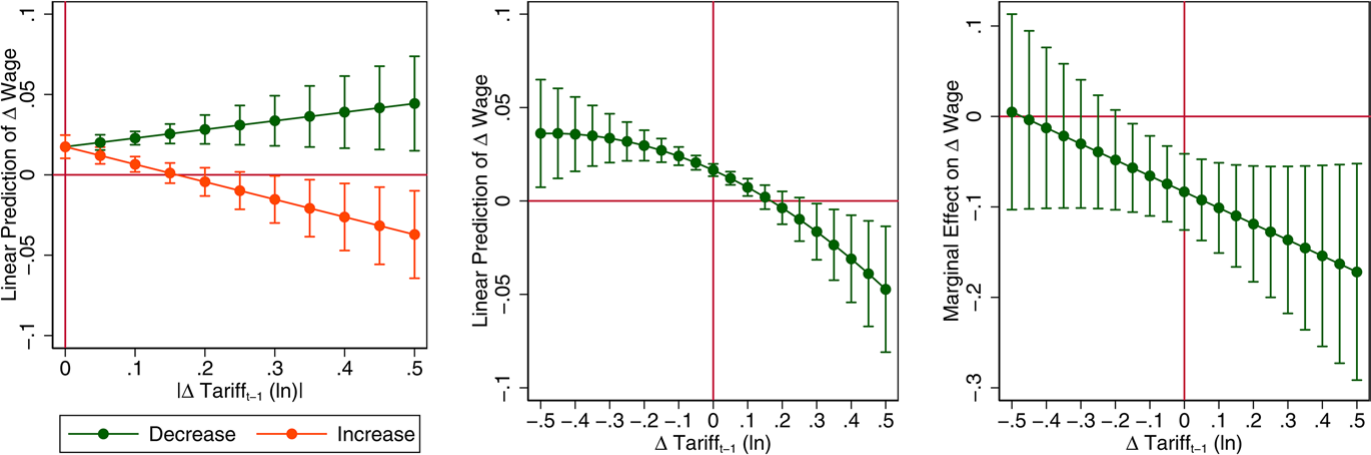
The asymmetric effect on wages of tariff increase and decreases

**Fig. 4:**
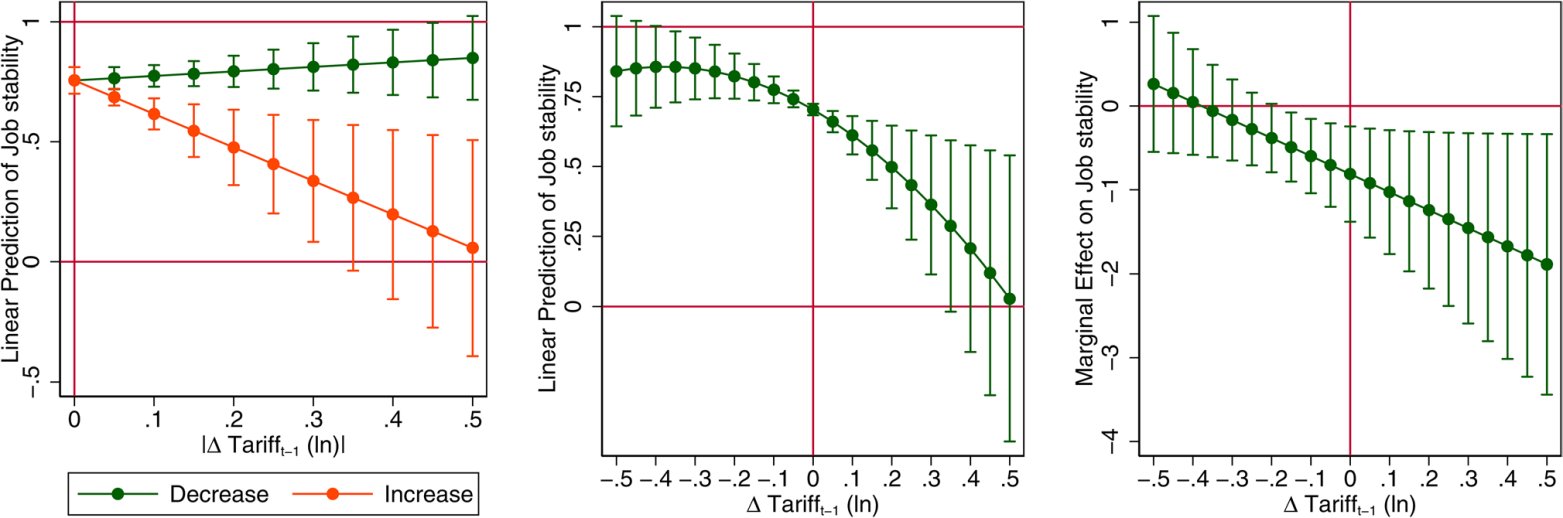
The asymmetric effect on job stability of tariff increase and decreases

As highlighted in the first part of the paper, during the last two decades, tariffs changes were not unidirectional nor smooth: the liberalization period was followed by a return to protectionism, and even during the liberalization period tariff reductions were not uniform with even some tariff increases. We use this source of variability to uncover the potential asymmetric effects of tariff increases vis à vis tariff reductions. To this end, as discussed in the methodology section, we first decompose tariff changes into its positive and negative components and then we further allow for nonlinear effects by introducing a quadratic term. Since the effect of tariffs now comes from the interaction terms, results are more clearly visualized by looking at predictive margins and marginal effects. This is done is Fig. 3 for wages and in Fig. 4 for job stability. The full regression tables are reported in “Appendix” in Table 12. The left panel of Fig. 3 displays the predictive margins for wages from the model in which we separate tariff increases and decreases (see Models 1 and 2 of Table 12). Tariff liberalization and protection yield different outcomes: tariff reductions have a slightly positive effect on wages; however, this effect is not statistically different from zero; on the contrary, tariff increases reduce wages. While most of the literature treats tariff changes as symmetric, our results seem to support the idea that they are in fact asymmetric: a 1% increase in tariffs is associated with a 0.12% reduction in wages; on the contrary, a decrease in tariffs brings about only minor increases in wages or none at all (see Model 1 of Table 12). Results from the model allowing for a nonlinear effect of tariff changes confirm these findings. The central and right panels of Fig. 3 (obtained from Model 3 of Table 12) show that the negative effect of tariff changes on wages tends to become larger for tariff increases. Looking at the confidence intervals the magnitude of the effect has some degree of uncertainty, but the effect is negative for tariff increases, while it is indistinguishable from zero for tariff decreases below 10% approximately. Very similar findings apply to job stability, as shown Fig. 4 (see also Model 4, 5 and 6 in Table 12).^30^ This implies that tariff liberalization, even if relatively large, do not seem to negatively impact workers’ wages nor job stability, while protectionism is harmful as it tends to reduce wages and make job positions more unstable.

We now consider possible unequal effects of tariffs on skill and gender. Results for the variables of interest are reported in Table 10 (Table 13 reports the coefficients of all the regressors). The interaction terms in Models 1 and 2 are insignificant suggesting that we are unable to detect an unequal effect of tariff changes on wages, either by skill or gender.

**Table 10:** Unequal effects by skill and gender, coefficients of interest

Variables	(1)	(2)	(3)	(4)
	Δ Wage	Δ Wage	Job stability	Job stability
Δ Tariff_t-1_	− 0.098***	− 0.102	− 0.352***	− 0.715
	(0.029)	(0.088)	(0.113)	(0.542)
Δ Tariff_t-1_ (ln) × blue collar	0.008		− 0.476*	
	(0.040)		(0.246)	
Δ Tariff_t-1_ (ln) × male		0.012		− 0.061
		(0.093)		(0.325)
Blue collar	0.002	0.002	− 0.050	− 0.041
	(0.008)	(0.009)	(0.037)	(0.034)
Male	0.017	0.018	− 0.101	− 0.110
	(0.011)	(0.015)	(0.080)	(0.109)
*(other regressors omitted)*				
Observations	1525	1525	3706	3706
R-squared	0.097	0.097	0.207	0.204
Governorate f.e. [22]	Yes	Yes	Yes	Yes
3-Digit sector f.e. [64]	Yes	Yes	Yes	Yes

Robust standard errors in parentheses. S.E. are clustered at the 3-digit sector level

*p < 0.1; **p < 0.05; ***p < 0.01

Tariff increase has a stronger negative effect on job stability of blue collars, while no asymmetric effect is found for gender. Note that in the gender models (i.e. Models 2 and 4) although the coefficient of tariff changes becomes insignificant, its marginal effect is negative and significant, consistently with other estimations. All in all, while we find that tariff increases and decreases yield asymmetric effects, our results—possibly also due to data constraints^31^—do not allow us to detect the existence of asymmetries by skill and gender, except for the case of unskilled workers whose job stability seems more negatively impacted by protectionist measures.

## Conclusion

During the 1990s, as several other developing economies and first among the MENA countries, Egypt undertook a policy of trade liberalization and concurrent labour market reforms. Despite large tariff reductions, which increased the openness of the country, Egypt remained one of the most protected economies of the area. At the same time, the reforms were not enough to address all the structural issues of Egypt’s labour market and yielded mixed results. More recently, after the 2008 financial crisis and the 2011 Spring Revolution, the country reverted to protection. Whether and to what extent tariff changes alleviated or worsened Egyptian workers’ conditions is an open question with relevant policy implications.

In this paper we investigate the possible effects of tariff reforms on labour market outcomes. Thanks to the recently released 2018 ELMPS, merging the available four survey waves with 3-digit tariff data from WITS, we are able to investigate the relation between tariff changes and workers’ wages and job stability over the 1998–2018 period, therefore performing de facto a long run analysis of this relationship to check if workers are hurt or have gained from different tariff policies and if different categories of workers are penalised/favoured in different way.

As compared to the wide theoretical and empirical literature on the relation between openness and the labour market (Harrison et al. 2011), few studies focused on Egypt, highlighting a negative correlation between tariffs and labour market outcomes, with unequal effects among workers (Zaki 2014). We contribute to this strand of the literature by analysing a longer time span including recently released data and, most importantly, investigating the possible asymmetric effects of tariff increases and decreases.

Our findings suggest that protectionist measures, implemented also in recent years, may have failed to protect workers and did not bring benefits in terms of labour market outcomes: tariff increases were correlated with reductions in the workers’ real wages and with a lower probability of having a permanent position. Furthermore, the effects of tariff changes are likely to be asymmetric: tariff increases and decreases, as expected, bring about opposite effects, and of different magnitude. The negative overall effect of tariff changes, consistently with the (scant) literature on Egypt, suggests that tariffs do not protect workers. Additionally, the asymmetry of the effects implies that protectionism does more harm than liberalizations do good. Previous liberalization waves, strongly supported by international organizations, have not been followed by significant changes in workers’ real wages, or, if any, the effect was only mildly positive. On the contrary, tariff increases are associated with non-negligible wage reductions. If Egyptian workers’ conditions deteriorated after the crises, the main causes have probably not much to do with increased import competition and tariff reductions of the previous liberalization waves, but mainly with other internal and external factors. Based on our results, the subsequent tariff increases may have further worsened workers’ conditions.

However, the aggregate results may hide important sources of heterogeneity between the different type of workers in the different sectors. This heterogeneity has implications for wage inequality, which is another relevant aspect of the labour market that must be considered in policy evaluations. To partly address this issue, we investigated sector specificities, for two important sectors for Egypt: food and textile. Moreover, we checked the presence of asymmetries by skills and gender both for wages and job stability. Not surprisingly, white-collars (skilled) and male workers display a wage premium relative to blue-collars (unskilled) and females, other things equal. Tariff changes do not seem to affect these wage gaps, while they negatively affect job stability of blue-collars.

Overall, our findings support the view that protectionism tends to hamper working conditions as well as increase inequality, while liberalizations did not improve nor deteriorate them. Note however that several caveats can apply. First, despite the fact that our results hold across several specifications (including IV) and although trade liberalizations in Egypt followed the indications of international institutions and were implemented similarly across sectors, which should reduce the possibility of endogeneity (Salem and Zaki 2019), a strict causal interpretation is probably still not warranted at this stage, also due to data limitations. Moreover, our sample covers only part of the economy, i.e. the formal labour market and tradeable sectors, thus our results must be understood as partial equilibrium analysis focusing on the individual effects. Further studies should address a series of important general equilibrium aspects including, among others, possible cross-sectoral effects.

While our and previous results consistently point towards a negative correlation between tariffs and wages in Egypt, more research is required to appropriately draw precise policy indications. Yet, our findings have important policy implications. The evidence that tariff increases are negatively associated with subsequent deterioration in labour market outcomes of workers is at odds with the recent decision of the Egyptian government to protect some of its sectors, even if these measures may help reducing the country’s trade deficit. Furthermore, our results add to a recent one by Zarzoso et al. (2018), who find a negative association between tariffs and total factor productivity. Trade protection, thus, may hamper the (already low) internationalization of Egyptian firms and participation into global value chains, slowing down the growth of more productive firms and worsening workers’ conditions.

## Figures and Tables

**Fig. 5:**
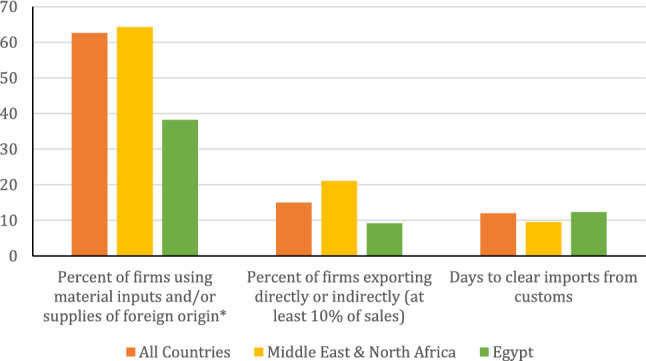
Indicators for Egypt’s participation to international trade. Note: “All countries” averages of indicators are computed by taking a simple average of country-level point estimates. For each economy, only the latest available year of survey data is used in this computation. *Data only for the manufacturing sector (Source: World Bank Enterprise Surveys 2016)

**Fig. 6:**
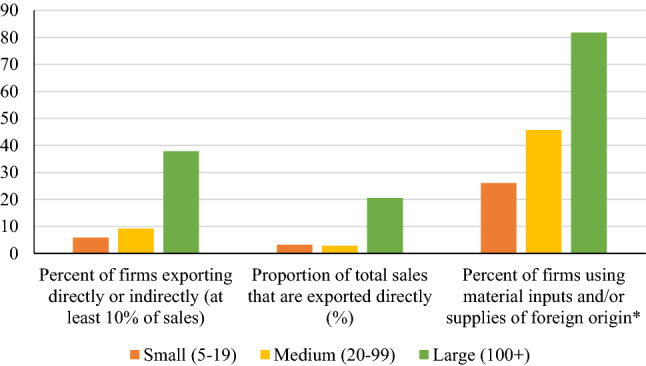
Indicators for participation to international trade, by firm size. Note: For each economy, only the latest available year of survey data is used in this computation. *Data only for the manufacturing sector (Source: World Bank Enterprise Surveys 2016)

## Appendix

See Tables 11.

**Table 11:** Sectoral composition of workers

Industry	N of workers
Agriculture	20,170
Mining	100
Manufacturing	5862
Food, beverages and tobacco	618
Textiles and apparel	594
Leather and related products	577
Paper, paper products and wood	271
Printing and reproduction of recorded media	358
Coke and refined petroleum products	250
Chemicals and chemical products	258
Pharmaceutical products	113
Rubber and plastics products	104
Other non-metallic mineral products	408
Basic metals	205
Fabricated metal products, except machinery and equipment	314
Computer, electronic and optical products	192
Electrical equipment	137
Machinery and equipment n.e.c	199
Motor vehicles, trailers and other transportation equipment	166
Furniture	645
Other manufacturing	31
Repair and installation of machinery and equipment	60
Services	399
Total	32,031

### Further econometric results

See Tables 12, 13, 14, 15 and 16.

**Table 12:** The asymmetric effects of tariff increase and decreases (full table)

Variables	(1)	(2)	(3)	(4)	(5)	(6)
	Δ Wage	Δ Wage	Δ Wage	Job stability	Job stability	Job stability
|Δ Tariff_t-1_ (ln)| × Incr	− 0.124***	− 0.184***		− 1.396***	− 1.584***	
	(0.035)	(0.046)		(0.490)	(0.536)	
|Δ Tariff_t-1_ (ln)| × Decr	0.060			0.188		
	(0.039)			(0.201)		
|Δ Tariff_t-1_ (ln)|		0.060			0.188	
		(0.039)			(0.201)	
Δ Tariff_t-1_			− 0.094***			− 0.813***
			(0.024)			(0.284)
(Δ Tariff_t-1_)^2^			− 0.100*			− 1.076*
			(0.059)			(0.548)
Age	− 0.001***	− 0.001***	− 0.001***	0.005***	0.005***	0.005***
	(0.000)	(0.000)	(0.000)	(0.002)	(0.002)	(0.002)
Male	0.017	0.017	0.017	− 0.093	− 0.093	− 0.088
	(0.011)	(0.011)	(0.011)	(0.061)	(0.061)	(0.064)
Urban	0.009	0.009	0.009	0.019	0.019	0.021
	(0.008)	(0.008)	(0.008)	(0.035)	(0.035)	(0.034)
Married	0.002	0.002	0.003	0.035**	0.035**	0.034**
	(0.008)	(0.008)	(0.008)	(0.017)	(0.017)	(0.016)
Formal	− 0.001	− 0.001	− 0.001	0.123***	0.123***	0.126***
	(0.009)	(0.009)	(0.009)	(0.034)	(0.034)	(0.033)
SME	0.001	0.001	0.001	0.005	0.005	0.006
	(0.008)	(0.008)	(0.008)	(0.028)	(0.028)	(0.029)
POE	− 0.004	− 0.004	− 0.004	0.070**	0.070**	0.067**
	(0.013)	(0.013)	(0.013)	(0.033)	(0.033)	(0.033)
Union	0.034***	0.034***	0.033***	0.091***	0.091***	0.088***
	(0.011)	(0.011)	(0.011)	(0.024)	(0.024)	(0.023)
Foreign ownership	0.147**	0.147**	0.146**	0.403***	0.403***	0.422***
	(0.062)	(0.062)	(0.060)	(0.113)	(0.113)	(0.121)
Blue collar	0.002	0.002	0.002	− 0.045	− 0.045	− 0.045
	(0.009)	(0.009)	(0.009)	(0.034)	(0.034)	(0.034)
Constant	0.055**	0.055**	0.054**	0.539***	0.539***	0.471***
	(0.021)	(0.021)	(0.022)	(0.156)	(0.156)	(0.173)
Observations	1525	1525	1525	3706	3706	3706
R-squared	0.098	0.098	0.099	0.217	0.217	0.214
Governorate f.e. [22]	Yes	Yes	Yes	Yes	Yes	Yes
3-Digit sector f.e. [64]	Yes	Yes	Yes	Yes	Yes	Yes

Robust standard errors in parentheses. S.E. are clustered at the 3-digit sector level

*p < 0.1; **p < 0.05; ***p < 0.01

**Table 13:** Unequal effects by skill and gender (full table)

Variables	(1)	(2)	(3)	(4)
	Δ Wage	Δ Wage	Job stability	Job stability
Δ Tariff_t-1_	− 0.098***	− 0.102	− 0.352***	− 0.715
	(0.029)	(0.088)	(0.113)	(0.542)
Δ Tariff_t-1_ (ln) × blue collar	0.008		− 0.476*	
	(0.040)		(0.246)	
Δ Tariff_t-1_ (ln) × male		0.012		− 0.061
		(0.093)		(0.325)
Blue collar	0.002	0.002	− 0.050	− 0.041
	(0.008)	(0.009)	(0.037)	(0.034)
Male	0.017	0.018	− 0.101	− 0.110
	(0.011)	(0.015)	(0.080)	(0.109)
Age	− 0.001***	− 0.001***	0.006***	0.006***
	(0.000)	(0.000)	(0.002)	(0.002)
Urban	0.009	0.009	0.021	0.020
	(0.008)	(0.008)	(0.033)	(0.033)
Married	0.002	0.002	0.036**	0.034*
	(0.008)	(0.008)	(0.017)	(0.017)
Formal	− 0.000	− 0.000	0.127***	0.123***
	(0.009)	(0.009)	(0.035)	(0.036)
SME	0.001	0.001	0.019	0.013
	(0.008)	(0.008)	(0.030)	(0.029)
POE	− 0.005	− 0.005	0.057	0.065*
	(0.013)	(0.013)	(0.034)	(0.035)
Union	0.033***	0.034***	0.107***	0.095***
	(0.011)	(0.011)	(0.026)	(0.025)
Foreign ownership	0.152**	0.151**	0.472***	0.487***
	(0.062)	(0.061)	(0.112)	(0.106)
Constant	0.048**	0.047**	0.429**	0.437**
	(0.023)	(0.020)	(0.189)	(0.209)
Observations	1525	1525	3706	3706
R-squared	0.097	0.097	0.207	0.204
Governorate f.e. [22]	Yes	Yes	Yes	Yes
3-Digit sector f.e. [64]	Yes	Yes	Yes	Yes

Robust standard errors in parentheses. S.E. are clustered at the 3-digit sector level

*p < 0.1; **p < 0.05; ***p < 0.01

**Table 14:** Wage regressions, food and textile specificity

Variables	(1)	(2)	(3)	(4)	(5)
	All sectors excl. food	Food only	All sectors excl. textile	Textile only	All sectors with food and textile interactions
	Real wage (ln)	Real wage (ln)	Real wage (ln)	Real wage (ln)	Real wage (ln)
Tariff_t-1_ (ln)	− 0.123***	− 0.023	− 0.104***	− 0.180	− 0.126***
	(0.017)	(0.036)	(0.027)	(0.094)	(0.018)
Tariff_t-1_ (ln) × food					0.107***
					(0.040)
Tariff_t-1_ (ln) × textile					0.049
					(0.074)
Age	0.003**	0.008***	0.003***	0.008	0.004***
	(0.001)	(0.002)	(0.001)	(0.006)	(0.001)
Male	0.215***	0.283**	0.210***	0.219***	0.224***
	(0.026)	(0.095)	(0.027)	(0.025)	(0.026)
Urban	0.017	0.063	0.025	0.058	0.027
	(0.026)	(0.067)	(0.024)	(0.091)	(0.023)
Married	0.150***	0.132*	0.140***	0.282**	0.148***
	(0.032)	(0.050)	(0.027)	(0.075)	(0.028)
Formal	0.116***	0.092	0.107***	0.145**	0.115***
	(0.024)	(0.049)	(0.021)	(0.056)	(0.020)
SME	− 0.012	− 0.009	− 0.028	0.050	− 0.015
	(0.037)	(0.052)	(0.038)	(0.031)	(0.032)
POE	0.020	0.053	0.047	− 0.102*	0.032
	(0.035)	(0.086)	(0.032)	(0.040)	(0.033)
Union	0.294***	0.260**	0.296***	0.186***	0.289***
	(0.031)	(0.082)	(0.031)	(0.045)	(0.029)
Foreign ownership	0.921***	0.233	0.675**		0.699***
	(0.203)	(0.147)	(0.254)		(0.243)
Blue collar	− 0.148***	− 0.107*	− 0.149***	− 0.097	− 0.144***
	(0.023)	(0.043)	(0.021)	(0.077)	(0.021)
Constant	2.217***	1.656***	2.183***	2.100***	2.194***
	(0.103)	(0.045)	(0.111)	(0.309)	(0.097)
Observations	6423	688	6646	465	7111
R-squared	0.240	0.219	0.223	0.378	0.237
Governorate f.e. [22]	Yes	Yes	Yes	Yes	Yes
3-Digit sector f.e. [64]	Yes	Yes	Yes	Yes	Yes

Robust standard errors in parentheses. S.E. are clustered at the 3-digit sector level

*p < 0.1; **p < 0.05; ***p < 0.01

**Table 15:** Wage regressions, subperiods

Variables	(1)	(2)	(3)	(4)
	98–12 Real wage	12–18 Real wage	98–12 Δ Wage	12–18 Δ Wage
Tariff_t-1_ (ln)	− 0.181***	− 0.052**		
	(0.026)	(0.020)		
Δ Tariff_t-1_ (ln)			0.017	− 0.087***
			(0.037)	(0.025)
Age	0.006***	0.002**	− 0.001**	− 0.002***
	(0.002)	(0.001)	(0.000)	(0.001)
Sex	0.265***	0.152***	0.017	0.010
	(0.027)	(0.040)	(0.018)	(0.012)
Urban	0.025	0.030	0.001	0.010
	(0.024)	(0.033)	(0.014)	(0.011)
Married	0.129***	0.113***	− 0.000	0.009
	(0.033)	(0.031)	(0.011)	(0.011)
Formal	0.110***	0.133***	0.016	− 0.004
	(0.029)	(0.027)	(0.012)	(0.011)
SME	− 0.039	0.013	− 0.004	0.001
	(0.039)	(0.033)	(0.011)	(0.010)
POE	− 0.000	0.018	− 0.026*	0.006
	(0.053)	(0.032)	(0.014)	(0.014)
Union	0.290***	0.286***	0.032***	0.024*
	(0.035)	(0.038)	(0.011)	(0.014)
Foreign ownership	0.712**	0.305	0.218***	0.146**
	(0.273)	(0.306)	(0.042)	(0.057)
Blue collar	− 0.187***	− 0.125***	0.018	0.006
	(0.033)	(0.020)	(0.013)	(0.010)
Constant	2.151***	2.226***	0.051	0.053*
	(0.125)	(0.113)	(0.037)	(0.029)
Observations	4504	4975	841	1264
R-squared	0.294	0.209	0.124	0.103
Governorate f.e. [22]	Yes	Yes	Yes	Yes
3-Digit sector f.e. [64]	Yes	Yes	Yes	Yes

Robust standard errors in parentheses. S.E. are clustered at the 3-digit sector level

*p < 0.1; **p < 0.05; ***p < 0.01

**Table 16:** Regression with year fixed effects, governorate trend and sectoral trend

Variables	(1)	(2)	(3)	(4)	(5)
	Real wage (ln)	Real wage (ln)	Real wage (ln)	Real wage (ln)	Real wage (ln)
Tariff_t-1_ (ln)	− 0.030***	− 0.035***	− 0.018**	− 0.048***	− 0.051***
	(0.003)	(0.004)	(0.007)	(0.010)	(0.007)
Age	0.004*	0.004*	0.004*	0.003	0.003
	(0.002)	(0.002)	(0.002)	(0.002)	(0.002)
Male	0.282***	0.264***	0.262***	0.273***	0.268***
	(0.049)	(0.055)	(0.055)	(0.055)	(0.053)
Urban	0.063***	0.015	0.027	0.023	0.025*
	(0.007)	(0.010)	(0.014)	(0.017)	(0.011)
Married	0.138**	0.151**	0.151**	0.155**	0.154**
	(0.040)	(0.046)	(0.047)	(0.048)	(0.050)
SME	− 0.029	− 0.026	− 0.039	− 0.045	− 0.039
	(0.021)	(0.029)	(0.030)	(0.036)	(0.025)
POE	0.096***	0.090***	0.086***	0.102***	0.110***
	(0.020)	(0.016)	(0.014)	(0.019)	(0.021)
Union	0.348***	0.335***	0.331***	0.333***	0.338***
	(0.016)	(0.013)	(0.012)	(0.013)	(0.010)
Foreign ownership	0.752***	0.762***	0.769***	0.721***	0.729***
	(0.011)	(0.013)	(0.004)	(0.018)	(0.016)
Blue collar	− 0.192***	− 0.181***	− 0.187***	− 0.175***	− 0.176***
	(0.006)	(0.007)	(0.006)	(0.006)	(0.013)
Constant	1.761***	1.949***	1.964***	1.921***	2.044***
	(0.066)	(0.084)	(0.053)	(0.124)	(0.149)
Observations	7163	7163	7163	7163	7163
R-squared	0.183	0.217	0.219	0.213	0.222
Year f.e. [4]	Yes	Yes	Yes	No	No
Governorate f.e. [22]	No	Yes	Yes	No	No
1-Digit sector f.e. [7]	No	No	Yes	No	No
Sectoral trend	No	No	No	Yes	No
Governorate trend	No	No	No	No	Yes

Robust standard errors in parentheses. S.E. are clustered at the 1-digit sector level

*p < 0.1; **p < 0.05; ***p < 0.01

### Further information on trade and trade policy in Egypt

*The information reported in this section provides some additional details that complement the discussion on Egypt’s trade policy reforms done in Sect.* *3* *of the paper.*

The liberalization policies followed in Egypt in the 1990s increased the (low) international projection of the economy, even though import protection remained high relative to other countries. Exports more than doubled in quantity, with an increase of 135%, from $4.4 billion in 1998 to $10.5 in 2003–2004. Moreover, if in 2005 Egypt’s main export were fuel products, during the trade liberalization phase Egypt’s economy became more diversified, with fuel exports declining to 29.3% of total exports in 2011 and to 14.3% in 2016 (WTO 2018). Despite these developments, for the entire period, the Egyptian trade balance was in deficit. The strong need of raw materials, semi-finished products and investment goods made the volume of imports exceed the volume of exports.

If before 2011 the Egyptian government was committed towards a reduction in the number of tariff bands and to tariff cuts, in the aftermath of the 2011 Revolution, it changed direction. To face various economic challenges, among which also a widening of the trade deficit, the Egyptian government raised import tariffs on a wide range of products, mainly non-agricultural goods (WTO 2018), including electronic devices, clothing, shoes, household appliances and plastics. In December 2016, it raised import tariffs for 364 tariff lines. In the Presidential Decree, the government described these goods as “provocative” or “unnecessary”. For most products, the increase was between 100 and 200%, while for the others it varied between 50, 125, 300, 500 and 700%.^32^ The lift on import tariffs aimed at cutting $49 bn trade deficit.^33^

To date, the top export destinations of Egypt are the United Arab Emirates ($2.69B), Italy ($2.02B), Turkey ($1.98B), the United States ($1.69B) and Germany ($1.51B). The top import origins are China ($8.07B), Russia ($5.84B), Germany ($3.45B), the United States ($3.38B) and Italy ($3.19B). Data from 2017 show that Egyptian exports are mainly of crude petroleum (15.6%), gold (9.2%), nitrogenous fertilizers (3.8%), refined petroleum (3.0%) and insulated wire (3.0%). Imports instead include wheat (6.5%), petroleum gas (5.6%), semi-finished iron (3.1%) and crude petroleum (2.5%) (OEC 2019).^34^

The intensity of the foreign trade of the private sector is definitely below the average of the Middle East and North African countries and generally below the world’s average, as we can perceive from the extremely low percentage of firms that directly or indirectly export and the percentage of firms using inputs of foreign origins (Fig. 5). Compared to the rest of region, Egyptian firms face notable inefficiencies regarding customs procedures: e.g., it takes almost 13 days to clear imports from customs, against the nine for the whole MENA area. These figures clearly show that there is room to improve Egypt’s position in the rankings on ease of doing business or on trade facilitation aspects.

The micro, small and medium enterprises (MSME) sector appears to have particularly weak exporting capabilities when compared to the whole Egyptian economic panorama. As it is natural to expect, and Fig. 6 shows, only 5% of small and 9% of the medium firms directly or indirectly export, but the share increases with the size of the firms, with almost 38% of large firms engaged in exporting activities.

## Footnotes

1 Public consensus towards more protective measures gradually increased in many countries after the 2008 financial crisis. The recent US-China trade war comes after a long debate, even among scholars, on the possible crowding-out of US workers due to Chinese imports (Autor et al. 2013). Despite the general view among economists, supported by facts, is that protectionist measures are costly even to the imposing countries (Amiti et al. 2019; Fajgelbaum et al. 2020) in the last months the Covid-19 outbreak gave new voice in support of trade restrictions such as export bans (see, for instance, the Financial Times article “Covid-19 is bringing out protectionist instincts”, April 19th 2020 (available at https://www.ft.com/content/ed78b09c-80a3-11ea-8fdb-7ec06edeef84). See also the https://macmap.org/covid19 to have a clear picture of the restrictive measures recently implemented.

2 In the choice among the several available indicators for trade liberalization, we refer to Goldberg and Pavcnik (2004). Indeed, the authors argue that the use of tariffs provides advantages over other measures, such as degree of openness of the economy, as they can be comparable across time and vary substantially across industries during trade reforms. However, there may be concerns, since tariffs depend strongly on the specific country and its use of tariffs. The issue does not have a definite answer. We use tariffs in the following analysis.

3 See for instance Alissa (2007).

4 See also the World Inequality Database (available at https://wid.world/country/egypt/).

5 There might be, however, important sources of heterogeneity by sector. For instance, some sectors had specific shocks due technological change or foreign competition, as we discuss later, or may be more or less affected by tariff changes. In thus study, we control for unobserved sectoral characteristics with industry-specific f.e., but we do not perform an ad hoc sectoral analysis, which is outside the scope of this paper. Nonetheless, we briefly consider separately (see “Appendix”) two important sectors: Food and Beverages and Textile. Results points towards the possible existence of sectoral specificities in the effect of tariffs; however, the general result that tariffs negatively affect wages of Egyptian workers seem to hold.

6 We confine the review of the literature to articles focused on wages and job stability. The amount of recent papers on these issues is very large and it is outside the scope of this paper to review all the possible mechanisms. See Harrison et al. (2011) for a comprehensive survey.

7 See for instance Dutta (2007) for a longer description of this mechanism for a developing country (in this case India).

8 Martins and Opromolla (2009) find that productivity increases systematically when firms engage in either exporting and importing, with positive repercussions also on wages.

9 For extensive and comprehensive reviews of both the theoretical and empirical evidence on trade policy and wages, see Goldberg and Pavnick (2007) and Helpman et al. (2010b).

10 Moreover, over the 1998–2018 period, the Egyptian pound (EGP) went through a large depreciation from 3.4 to 17.8 EGP per USD. The depreciation was not smooth:2016 was marked by a strong and fast devaluation with the currency passing from 8.9 to 18.7 EGP per USD. The 2016 devaluation reinforced the tariff increases in order to discourage imports. These macro trends clearly had an impact on the Egyptian economy and on its workers. However, as we argue in the paper, these macroeconomic trends do not seem to drive our estimates.

11 See https://ec.europa.eu/trade/policy/countries-and-regions/regions/euro-mediterranean-partnership/.

12 For a complete review of all the trade agreements signed by Egypt, see the Trade Policy Review of Egypt 2018 of the WTO (https://www.wto.org/english/tratop_e/tpr_e/tp467_e.htm). For the AfcFTA, see the World Economic Forum article “Africa’s new free trade area is promising, yet full of hurdles” (available at https://www.weforum.org/agenda/2019/09/africa-just-launched-the-world-s-largest-free-trade-area/) and the CNBC article “Africa is on the verge of forming the largest free trade area since the World Trade Organization” by Crabtree, March 20, 2018 (available at: https://www.cnbc.com/2018/03/20/africa-leaders-to-form-largest-free-trade-area-since-the-wto.html and https://au.int/en/cfta).

13 See the US International Trade Administration article on Egypt’s import tariffs (available at https://www.export.gov/apex/article2?id=Egypt-Import-Tariffs).

14 As reported by Reuters (at https://www.reuters.com/article/us-egypt-economy-tariffs-idUSKBN13T0DN) and by the Middle East Eye (at https://www.middleeasteye.net/news/egypt-raises-tariffs-imported-goods-60-percent-692827788).

15 Several studies found, before the 25th of January Egyptian revolution in 2011, a positive correlation between the presidential election year and the increase in the percentage of special bonuses that should be added to the basic wage.

16 As reported by the ILO (see https://www.ilo.org/dyn/normlex/en/f?p=NORMLEXPUB:13101:0::NO::P13101_COMMENT_ID:2699851).

17 See https://theforum.erf.org.eg/2019/04/25/raising-egypts-minimum-wage-impact-inequality/).

18 In this regard, the 2003 Labour Law must be mentioned. In order to loosen the existing legislation that was rather stringent both for employers and employees, in July 2003 the labour law No. 12 was passed, with the goal of increasing flexibility for employers in the private sector for hiring and firing their employees. Indeed, Wahba and Assad (2017) proved that the introduction of this law had a positive impact on the incidence of contracted jobs, thereby increasing formal employment. The share of formal employment however is still decreasing while that of irregular and informal employment are estimated at 40% and 30% respectively (ILO 2018).

19 Note that this is the same source of data of the aggregate tariff figures discussed in the first part of the paper.

20 It is also worth noting that Egypt grants at least MFN treatment to all WTO Members.

21 A different methodology has also been used in the literature. It employs a two-stage estimation strategy (see for instance Feliciano 2001; Kumar and Mishra 2008) where in the first equation the individual salary is regressed on individual characteristics and on an industry dummy for each year separately. Then, the industry wage premium is regressed on a set of industry-specific characteristics, among which also the indicator for trade liberalization. As highlighted in Murakami (2020) adopting this approach rules out the possibility to interact individual characteristics with sector-specific tariff levels. Moreover, the second stage estimation may suffer from small sample bias.

22 Specifically, $\tau =\mathrm{ln}(tariff+1)$. This a common definition, for instance used in Murakami (2020) and Di Comite et al. (2018) among others. This definition implies that the coefficients can be immediately read as elasticities. Moreover, it is particularly useful when tariff rate levels are very different between sectors, as in Egypt, because it allows to consider that, for instance, a 1 p.p. tariff rate increase provides different additional protection depending on the initial tariff rate level.

23 Although not shown in the paper but available on request, we run the baseline and first-difference regressions including among the regressors the initial level of tariffs (in 1998) and the log of imports. The inclusion of these two controls does not alter our results.

24 In other versions of our regressions, not reported in the paper but available on request, we also include the lagged dependent variables among our regressors as well as imports, with no significant alteration of the results.

25 On this issue see the seminal paper by Grossman and Helpman (1994) and the vast following theoretical and empirical literature. More recently, Annicchiarico and Marvasi (2019) generalized the results showing how they hold under more general conditions even for monopolistically competitive sectors with variable elasticity of substitution and when tariff pass-through is incomplete.

26 To ensure that our results do not depend on few extreme values or misreporting errors, the results presented in this section exclude outliers for our key variables, namely wages and tariffs (i.e. observations below the 1st and above the 99th percentiles). The exclusion of outliers does not significantly affect the results.

27 In the panel estimations all the time-invariant regressors are absorbed by worker fixed effects. This applies, for instance, to the gender dummy, which is then dropped, and to governorate and sector dummies, although not perfectly due to a small fraction of individuals moving. Moreover, the fact that workers are not perfectly nested within sectors prevents the use of clustered standard errors in the panel estimations. As a check, we repeated the panel estimation with 3-digit sector fixed effect and clustered standard errors on workers that did not change sector, with consistent results.

28 As mentioned in the data description, the industry composition of the survey sample is skewed towards agriculture and manufacturing. As a further control, we checked our results on agriculture and manufacturing separately. Results are consistent and the coefficient of tariff remains negative and significant.

29 A linear probability model (LPM) presents some advantages as well as disadvantages. In the context of panel data or with endogenous dummies, as in our case, LPM is preferable to probit or logit (Angrist 2001; Angrist and Pischke 2008; Wooldrige 2002). The main disadvantage regards the risk of obtaining estimated probabilities outside the 0–1 range. Horrace and Oaxaca (2006) discuss the issue and propose a trimmed estimator that yields unbiased estimates. In our case, the issue seems of little practical relevance because the share of predicted probabilities outside the 0–1 interval is very low (about 5.6%). Moreover, the Horrace–Oaxaca trimming estimator yields similar or even stronger results. Finally, to be sure, we also estimated a probit model, with consistent results.

30 Note also that, as also discussed earlier the use of a linear probability model is supported by predictive margins lying within the (0, 1) interval for reasonable tariff changes.

31 To explore this possibility, we checked our results with less demanding specifications, i.e. by using 2-digit sector fixed effects and standard errors clustered at that level. Results indicate that relative to skilled workers, unskilled workers might be more negatively affected by tariff increases not only in terms of job stability but also in terms of wages, and that female workers may suffer more than males in terms of job stability. These results, however, may be driven by cross-sector characteristics not adequately taken into account by the specification used. It is possible that richer datasets might allow researchers to measure these unequal effects more precisely.
